# Eradicating rheumatic heart disease in Africa: have we made progress since the Drakensberg declaration?

**DOI:** 10.3389/fcvm.2024.1485501

**Published:** 2024-11-08

**Authors:** Mpiko Ntsekhe, Anton Doubell

**Affiliations:** ^1^ Division of Cardiology, Department of Medicine, Faculty of Medicine, University of Cape Town and Groote Schuur Hospital, Cape Town, South Africa; ^2^ Division of Cardiology, Department of Medicine, Faculty of Medicine and Health Sciences, Stellenbosch University and Tygerberg Hospital, Bellville, South Africa

**Keywords:** rheumatic heart disease, Africa, health system barriers, challenges and progress, pragmatic solutions

In 2006 the Drakensberg Declaration on the Control of Rheumatic Fever and Rheumatic Heart Disease in Africa (1) was published, launching the Awareness Surveillance Advocacy Prevention (A.S.A.P.) program to control rheumatic fever (RF) and rheumatic heart disease (RHD) in Africa. It is almost 20 years later and a good time to take stock of our progress in dealing with this scourge on the African continent. Two articles appearing in this issue of the Journal, a] serve as a reminder of the major knowledge and treatment gaps that remain in what is the most important preventable cause of valvular heart disease on the continent of Africa; and b] shed some light on areas of progress that are being made in the field to address them.

If the enormous morbidity and mortality that is still being caused by RHD in low- and middle-income countries is to be addressed, there is reasonable consensus that a multi-pronged attack (2–4) is required, including:

• Improvement in living conditions and health care programs in these communities
• Early detection and treatment of the precursors of RHD:
  ○ Group A beta-hemolytic streptococcal (GAS) pharyngitis
  ○ Acute rheumatic fever (ARF)
• Developing a protective vaccine to prevent GAS infection
• Early detection of RHD through screening programs
• Adherence to effective programs of prophylactic treatment
• Managing patients with advanced valve lesions requiring tertiary care and access to valve replacement intervention.
• Continuing research to fill the gaps in our knowledge regarding ARF and RHD

The poverty, poor housing and overcrowding present in most African countries, accompanied by a shortage of health care resources, are a reality that is likely to remain a challenge for some time to come. This reality has prevented many health programs across the continents from being able to diagnose and treat GAS and ARF, improve the detection of latent or early RHD, and administer prophylactic treatment effectively in order to prevent progression to symptomatic severe valve lesions and the debilitating complications such as heart failure that follow.

An important aspect of the RHD focused articles in this issue of the journal is that they remind us not to become overwhelmed by the enormity of the challenges preventing us from putting in place ideal solutions. The article by Sulafa et al, whose work focusses on developing reasonable strategies that may not be perfect solutions but are practical and implementable in disadvantaged communities is an example. In it the authors provide a simplified approach to manage GAS and ARF which may be particularly useful at primary and secondary care level where access to echocardiography and laboratory tests required for diagnosis is limited. Although developed specifically in and for Sudan, the algorithm for ARF management, is accompanied by detailed text on the administration of benzatine penicillin G (BPG) and can be put to use in many other resource-limited African countries with a similar ARF burden and health care limitation.

At the other end of the spectrum, the article from Egypt by Kotit and Jacoub in this issue provides insight into what is possible in Africa, with regard to management of advanced RHD at a tertiary centre with appropriate funding, infrastructure and expertise. Many of their patients presented with multivalvular or mitral valve disease requiring balloon valvuloplasty or surgical intervention and received their much needed percutaneous and surgical interventions with excellent results. However, the realization that very few countries on the African continent can provide these services (5) to those that need it serves to stress the need to identify latent or early RHD and prevent progression by administering prophylactic treatment.

The World Heart Federation (WHF) guidelines for the echocardiographic diagnosis of RHD, published in 2009 and updated in 2023 (6) are used by most workers in the field who are screening high risk populations in order to identify RHD early in order to initiate prophylactic treatment. Whilst these guidelines serve to drive a standardized approach to early case detection, there are significant limitations in this approach, including the complexity of the criteria and the expertise and equipment needed to assess these criteria. Efforts have been made to simplify these criteria such as the single parasternal-long-axis-view-sweep of the heart (SPLASH) in two dimensional (2D) and colour Doppler imaging proposed by Remenyi et al. (7). The WHF guidelines have largely developed from epidemiological data, expert opinion and end-user feedback and efforts to improve the performance of the diagnostic criteria have been hampered by the lack of a gold standard to diagnose RHD. A study by Hunter et al. published in 2023 addressed this limitation by developing a composite reference standard RHD-positive cohort ensuring the highest possible likelihood of RHD and a cohort with a very low risk RHD profile. They then developed morpho-mechanistic screening criteria that outperformed the WHF criteria to detect or rule out RHD in these two cohorts. More importantly they developed a simple two-step screening algorithm that will enable non-expert screeners to effectively screen for RHD in the field (8).

It is probably fair to say that, at the present time, we have not made enormous strides in Africa to eliminate RHD, a preventable cause of heart disease that has been all but eliminated in many high-income countries. It is likely that this will only be achieved against a backdrop of decreasing poverty, improving living conditions, improving education and developing well-funded, well-functioning health care programs. However, there is no need, until these objectives have been met, to not develop novel solutions and local solutions that may not be perfect but are feasible and practical in particular communities.
